# Extrapulmonary tuberculosis mortality according to clinical and point of care ultrasound features in Mozambique

**DOI:** 10.1038/s41598-022-21153-z

**Published:** 2022-10-05

**Authors:** Edy Nacarapa, Isabelle Munyangaju, Dulce Osório, Pereira Zindoga, Claudia Mutaquiha, Benedita Jose, Artur Macuacua, Bartolomeu Chongo, Marcelo de-Almeida, Maria-Elisa Verdu, Jose-Manuel Ramos-Rincon

**Affiliations:** 1 TB/HIV Division, Carmelo Hospital of Chókwè – The Daughters of Charity, Saint Vincent de Paul, Chókwè, Gaza Province Mozambique; 2 Tinpswalo Association, Research Unit, Vincentian Association to Fight AIDS and TB, Chókwè, Gaza Province Mozambique; 3 Macia Health Centre, Macia, Gaza Province Mozambique; 4 NTP – National Tuberculosis Program, Ministry of Health, Maputo, Mozambique; 5 Health District Directorate, Chókwè, Mozambique; 6 Internal Medicine Department, Alicante General University Hospital and University Miguel Hernandez de Elche, Elche, Spain

**Keywords:** HIV infections, Tuberculosis, Epidemiology, Outcomes research

## Abstract

In resource-limited settings, point-of-care ultrasound (POCUS) has great potential to support the timely diagnosis of extrapulmonary tuberculosis (EPTB). We aim to determine the in-hospital mortality due to EPTB according to clinical and POCUS features and risk factors in newly diagnosed patients hospitalized for EPTB in Chókwè district, Mozambique. We analyzed routinely collected data from paper medical files and electronic POCUS records of EPTB in infected patients aged 15 years or older and admitted to Carmelo Hospital of Chókwè from 2016 to 2020. Kaplan–Meier survival curves and adjusted Cox regression analyses were used to model predictors of mortality and time to death. The 390 included in-patients with EPTB and POCUS data contributed a total of 6240 in-hospital person-days of observation. The overall mortality rate was 2.16 per 100 person-days. Adjusted Cox regression showed a higher risk of death in those with abdominal tenderness (adjusted hazard ratio [aHR] 1.61, 95% confidence interval [CI] 1.00–2.82, p = 0.050), antiretroviral treatment (ART) for more than 90 days (aHR 4.03, 95% CI 1.50–10.78, p = 0.006), and mixed patterns on kidney POCUS (aHR 2.91, 95% CI 1.38–6.10, p = 0.005). An optimal immunovirological response to ART was a protective factor against death [aHR] 0.12, 95% CI 0.04–0.35, p < 0.001). Variables associated with an increased risk of death were male gender, abdominal pain, ART for more than three months (with immunovirological failure or non-response to ART) and having a mixed pattern of kidney POCUS characteristics. Early detection of these risk factors may have a direct impact on reducing TB mortality, and the POCUS approach as a complementary diagnostic method for EPTB provides a simple, feasible and affordable intervention in resource-limited settings like Mozambique.

## Introduction

Tuberculosis (TB) remains a severe public health problem and continues to contribute substantially to global morbidity and mortality. Mozambique is among the countries with the highest incidence of TB, and deaths during in-patient care continue to threaten the overall success of the Mozambican National TB Program (NTP) and the implementation of End TB Strategy (95–90 targets) by 2035^1^

The advent of affordable, portable ultrasound devices has led to increasing interest in the use of point-of-care ultrasound (POCUS), which has great potential to support the diagnosis of infectious diseases, especially in resource-limited settings^2^. The POCUS is a bedside ultrasound practical and fast, because it is patient-centered (does not require a specific room or complex referral procedures), it can elucidate important findings before microbiological results, allowing the physician to interpret images and make quick decisions, as opposed to the conventional ultrasound approach model, which requires an imaging department, senior staff, a specific performance room, equipment, and a patient referral procedure protocol^3,4^.

EPTB microbiological diagnosis is often difficult and requires high index of suspicion as the disease is paucibacillary and affects many anatomical sites other than the lung (with a variety of clinical presentations)^5^. The reference standard microbiological confirmation (by molecular, smear microscopy or culture) is often illusive in EPTB, clinical diagnosis is then complemented by cross-sectional imaging^2^.

POCUS has been widely used for the diagnosis of adult pneumonia with sensitivity and specificity having been comparable to that Chest X-ray, there is thus a belief that this success of POCUS in pneumonia could be seen as well for PTB; in a recent systematic review, although it is noted that there is still no sufficient evidence to determine the diagnostic accuracy of POCUS for PTB, the sensitivity and specificity ranged from 72.5–100.0% and 46.7–80.4% respectively for lung POCUS (for subpleural nodule and lung consolidation)^6^.

In case of any infection especially in pulmonary tuberculosis or EPTB, the definitive diagnostic-based confirmation comes from infective agent-specific detection via culturing, PCR or staining from a specimen taken from the patient (sputum, pus, CSF, pleural effusion, pericardial effusion, ascites, urine, biopsied tissue, etc.); meanwhile any diagnosis made other than by culture may only be classified as "probable" or "presumed"^7,8^. The big challenge to confirm TB infection from effusions fluid samples (Pleural, pericardial, and peritoneal) is low sensitivity and specificity; a Pakistan report of 21 ascites fluid samples analyzed showed sensitivity of GeneXpert was 28.57% and specificity was 0%^9^; an Indian report of 156 pleural fluid samples analyzed, Xpert assay has a very high specificity 100% in diagnosing tubercular pleural effusion but has a low sensitivity 16%^10^. The low sensitivity to confirm TB by microbiological reference standard (molecular, smear microscopy or culture) is often illusive in EPTB, clinical diagnosis is then complemented by POCUS, which can be used on top of those techniques not alone, especially in freshly infective cases.

In Eastern and Southern Africa, POCUS is increasingly being applied to support a timely diagnosis of pulmonary (PTB) and extrapulmonary tuberculosis (EPTB)^2,6^

Chókwè district has experienced a dramatic increase in the proportion of EPTB-infected people, from 18.2% in 2006 to 24.2% in 2017^4^, and it follows the NTP eligibility criteria to initiate TB treatment^11^. The standard diagnostic methods for EPTB before 2016 were based on clinical features associated with radiological and/or histological findings. In 2016, when the Carmelo Hospital of Chókwè (CHC) started using the POCUS, the proportion of EPTB in-patients increased^12^.

Although the POCUS represented a step forward in the CHC, concerns remain for the rest of the public sector care in Mozambique due to the lack of availability of POCUS for TB diagnosis. There is, therefore, a need for pragmatic research to clarify the impact of this method on EPTB diagnosis and treatment outcomes^2^. Such evidence is essential to inform the design of interventions to support implementation of POCUS for EPTB elsewhere.

This study aimed to determine the utility of POCUS in the diagnosis of EPTB and evaluate the in-hospital mortality due to EPTB according to clinical and POCUS features and risk factors in newly diagnosed patients hospitalized for EPTB in Chókwè district, Mozambique. Moreover, we aimed to determine whether the association between POCUS features of EPTB and mortality justifies implementation of POCUS in the Mozambican public health sector.

## Methods

### Study setting

Carmelo Hospital of Chókwè and its 26 primary healthcare clinics (PHC) serve the mainly rural Chókwè district in southern Gaza province^13^. The region has an area of approximately 1864 km^2^ and a Changana-speaking population of approximately 186,597. The hospital has 150 beds, with separate wards for internal medicine, pediatrics, TB, women, and men, and it is staffed by four general practitioners, working in the ward, under the command of a senior doctor, a specialist in TB, who assumes the clinical direction. Every year, the CHC handles approximately 10,000 outpatient visits and 1600 admissions with an average hospitalization of 24 days. The center specializes in TB/HIV and has been administered by Catholic missionaries (the Daughters of Charity, Saint Vincent de Paul) since 1993. It is responsible for TB screening and treatment, HIV testing, antiretroviral treatment (ART) initiation, management of in-patient and outpatient care, and monitoring of TB/HIV-positive patients. The available diagnostics are chest X-ray, POCUS, hematology, biochemistry, microbiology, parasitology, TB microscopy, Xpert MTB/RIF assay, urine TB-mycobacterial lipoarabinomannan (LAM), CD4 counts, and RNA HIV viral load. TB culture and histology are available but results rarely influence acute clinical management^14^. If required, patients can be referred to the Central Hospital of Maputo for further diagnostic assessment, such as computed tomography and magnetic resonance imaging scans, but this possibility is restricted by distance (about 250 km from the CHC) and limited access to appointments. Prevalence of HIV in adults aged 18 to 35 years is 29.4%^15^. In 2016, the CHC notified 817 cases of all forms of TB, of which 22.2% had EPTB and 33%, TB/HIV co-infection^12^. Health technicians or nurses start TB treatment for pulmonary forms; physicians diagnose smear-negative TB or EPTB based on clinical or radiological features according to current NTP guidelines^11^.

### Study design

**Figure 1:**
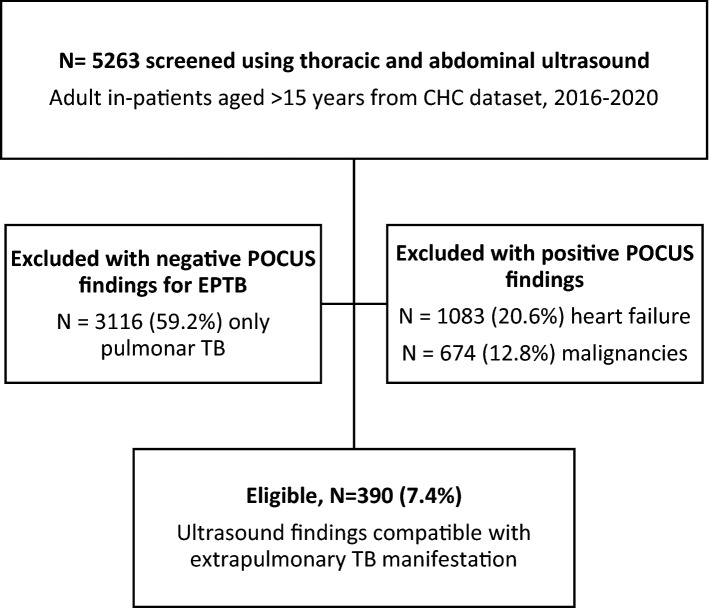
Extrapulmonary tuberculosis (TB) in-patient flowchart from Carmelo Hospital of Chókwè (CHC).

Patients were retrospectively enrolled into the study from January 2016 to December 2020. All adult (≥ 15 years) in-patients infected with EPTB were eligible based on the following inclusion criteria: three major clinical symptoms (fatigue, fever, night sweats ≥ 1 month), plus one or more minor symptoms (weight loss, peripheral adenopathy, abdominal tenderness, abdominal swelling, diarrhea > 1 month, dyspnea, and constipation); positive thoracic POCUS findings (neck and axillary adenopathy, pleural effusion, fibrinous pericardial effusion); and abdominal POCUS features (ascites, hepatomegaly, splenomegaly with focal lesion, para-aortic adenopathy > 1.5 cm, or renal abnormalities, including nephromegaly with hypo[anechoic] lesion, or atrophic renal fibrosis). Exclusion criteria were negative POCUS findings, exclusively pulmonary forms of TB without other organ involvement, and POCUS evidence of non-TB organic pathology (e.g., cirrhosis, congestive heart failure) (Fig. 1). The dataset was comprised of demographic variables, admission and discharge date, patient outcome (discharge or death), clinical symptoms, laboratory results (HIV status, CD4 cell count, HIV viral load, immunovirological response to ART on admission), chest X-ray, and thoracoabdominal POCUS reports.

There is no established ultrasound department at CHC; an experienced physician in thoracic and abdominal POCUS performed all exams using a SONOACE R3 v2.01.00-02 machine (released 25 March 2014, (https://www.samsungmedison.com), with a C2-4/20 convex probe and 1–10 MHz frequency.

#### Case definition according to POCUS findings

**Figure 2:**
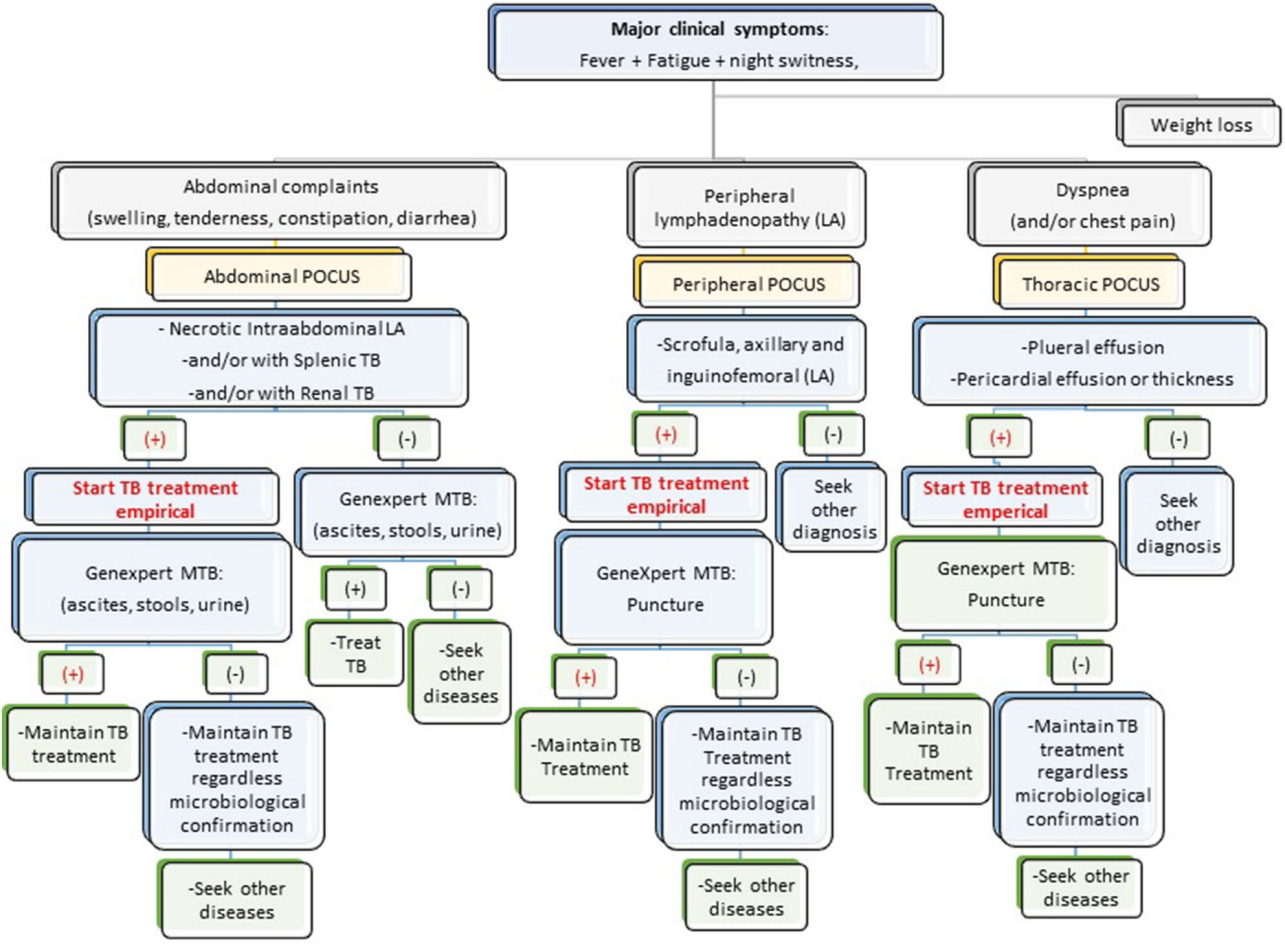
Point-of-Care Ultrasound (POCUS) algorithm adjusted to Carmelo Hospital of Chokwe for the management of extrapulmonary TB.

According to the original articles on ultrasound for TB diagnosis^16–23^, focused assessment with sonography for HIV/TB (FASH^24,25^ and POCUS^2,6,26^ algorithms tested in other countries, the adopted TB-defining sonographic findings were as follows (Fig. 2).

##### Peripheral tuberculous lymphadenitis

Peripheral tuberculous lymphadenitis (neck [scrofula], axillary or inguinofemoral TB) was diagnosed based on typical findings of lymph node enlargement greater than 1.5 cm (also known as lymphadenopathy, lymphadenitis, adenopathy, or adenitis). The following POCUS features were used to define tuberculous lymphadenitis: (1) on grey scale: rounded node, hypoechoic focus in deep subcutaneous tissues, nodal matting and surrounding soft tissue edema, nodal conglomeration forming masses^18^; and (2) on Doppler scale: present prominent hilar vascularity^16,17^. Fine-needle aspiration with Xpert MTB/RIF analysis for TB confirmation was performed.

##### Tuberculous pleural effusion

**Figure 3:**
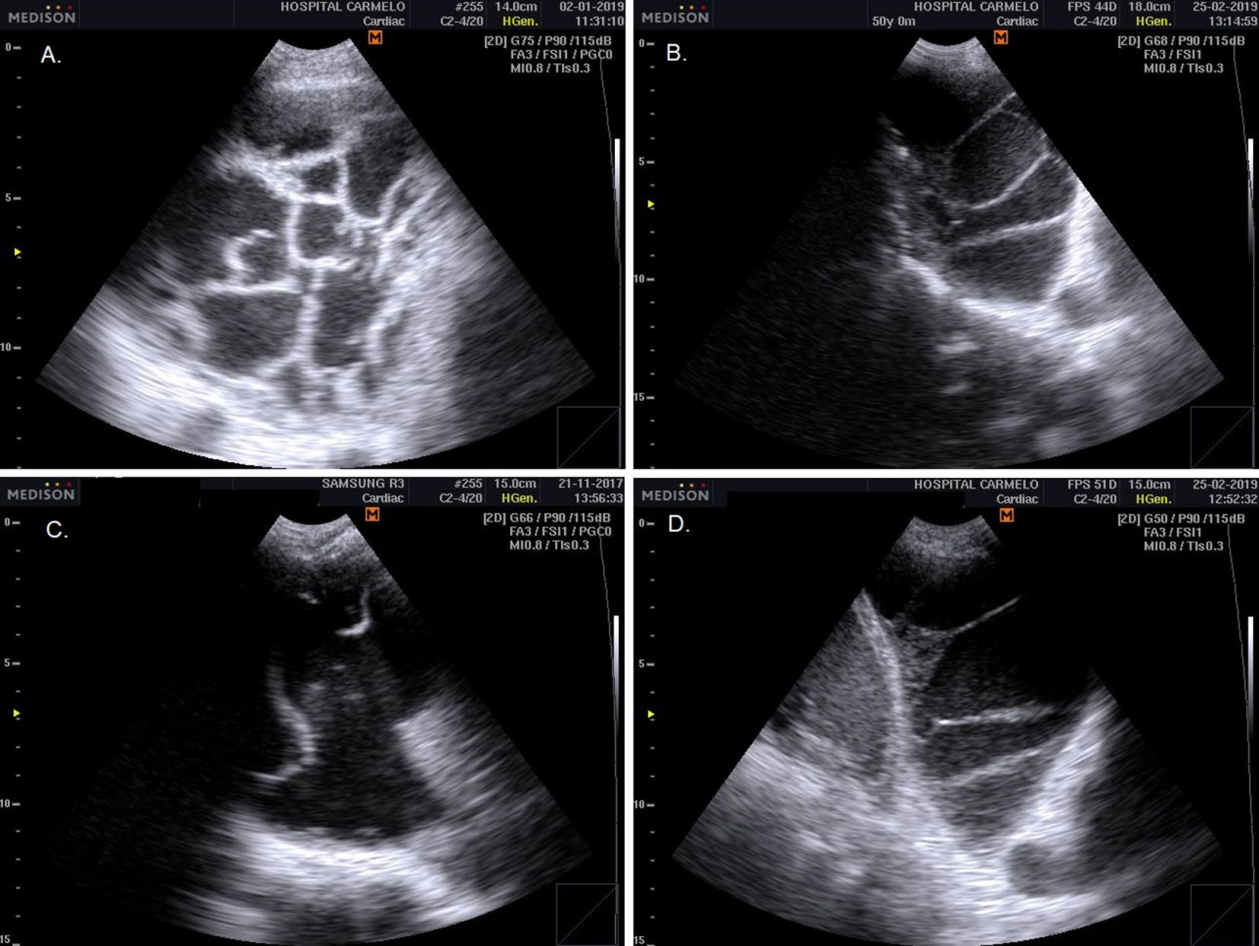
Pleural TB: pleural thickening adjoining a complex pleural effusion with multiple thin septation and fibrinous strands in the pleural space, producing a weblike or branching appearance.

Tuberculous pleural effusion was diagnosed based on pleural thickening adjoining a complex pleural effusion with multiple thin septation and fibrinous strands in the pleural space, producing a weblike or branching appearance^19^ (Fig. 3). Laboratory confirmation was not undertaken.

##### Pericardial tuberculous

**Figure 4:**
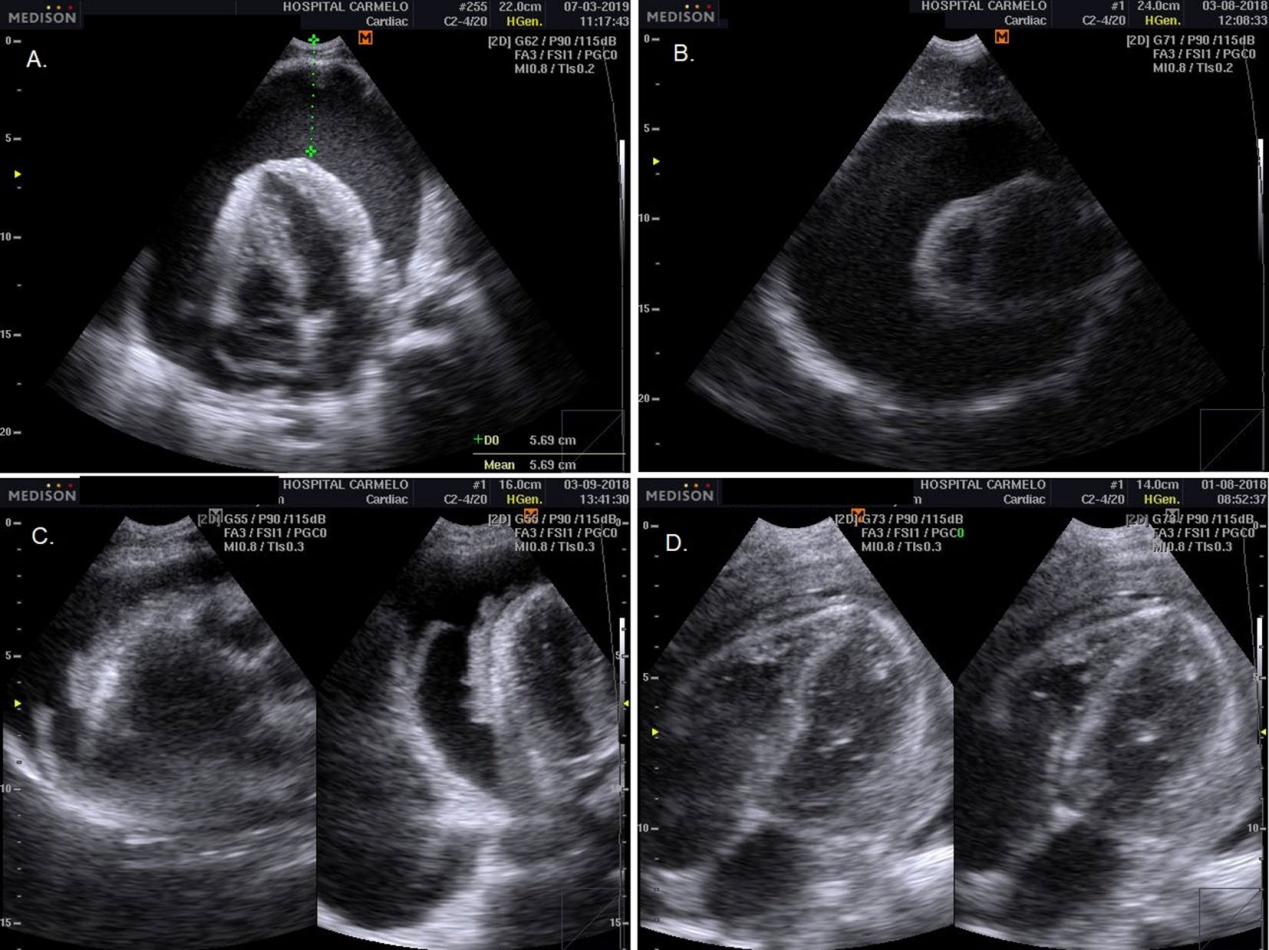
Pericardial TB: pericardial effusions with fibrinous filaments (**A**, **B**); and pericardial thickening (**C**, **D**) with complications, such as cardiac tamponade or impaired diastolic function, indicating pericardial constriction.

Pericardial TB was diagnosed either as: (1) based on pericardial effusions with fibrinous filaments; or (2) on pericardial thickening with complications, such as cardiac tamponade or impaired diastolic function, indicating pericardial constriction^20^ (Fig. 4). Laboratory confirmation was not performed.

##### Abdominal tuberculous

**Figure 5:**
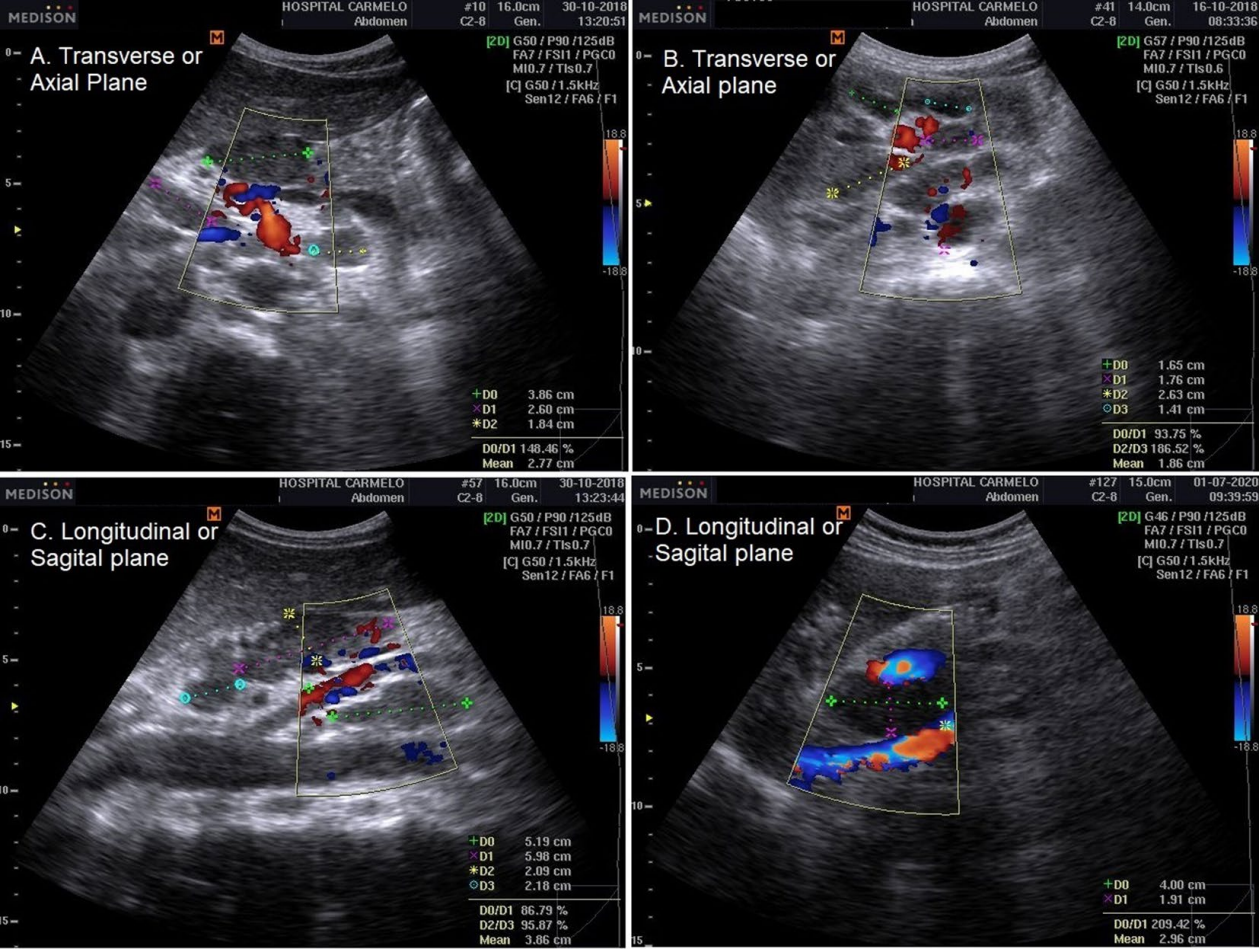
Intra-abdominal lymphadenitis TB: Conglomerate intra-abdominal (para-aortic, mesenteric) lymphadenopathy (lymph node enlargement greater than 1.5 cm; **A**– **C**); large hypoechoic lesion compatible with retrohepatic abscess (**D**).

Abdominal TB was diagnosed based on the presence of discrete or conglomerate intra-abdominal (para-aortic, mesenteric) lymphadenopathy (lymph nodes enlargement greater than 1.5 cm)^25^, regardless of additional supportive findings such as ascites, hepatomegaly, splenomegaly, and/or nephromegaly (Fig. 5). However, the manifestation of any additional findings not associated with intra-abdominal lymphadenitis was considered an exclusion criterion. Therefore, detailed analyses were performed to rule out other associated pathologies such as cirrhosis, congestive heart failure, nephrotic syndrome, or neoplastic disease.

**Figure 6:**
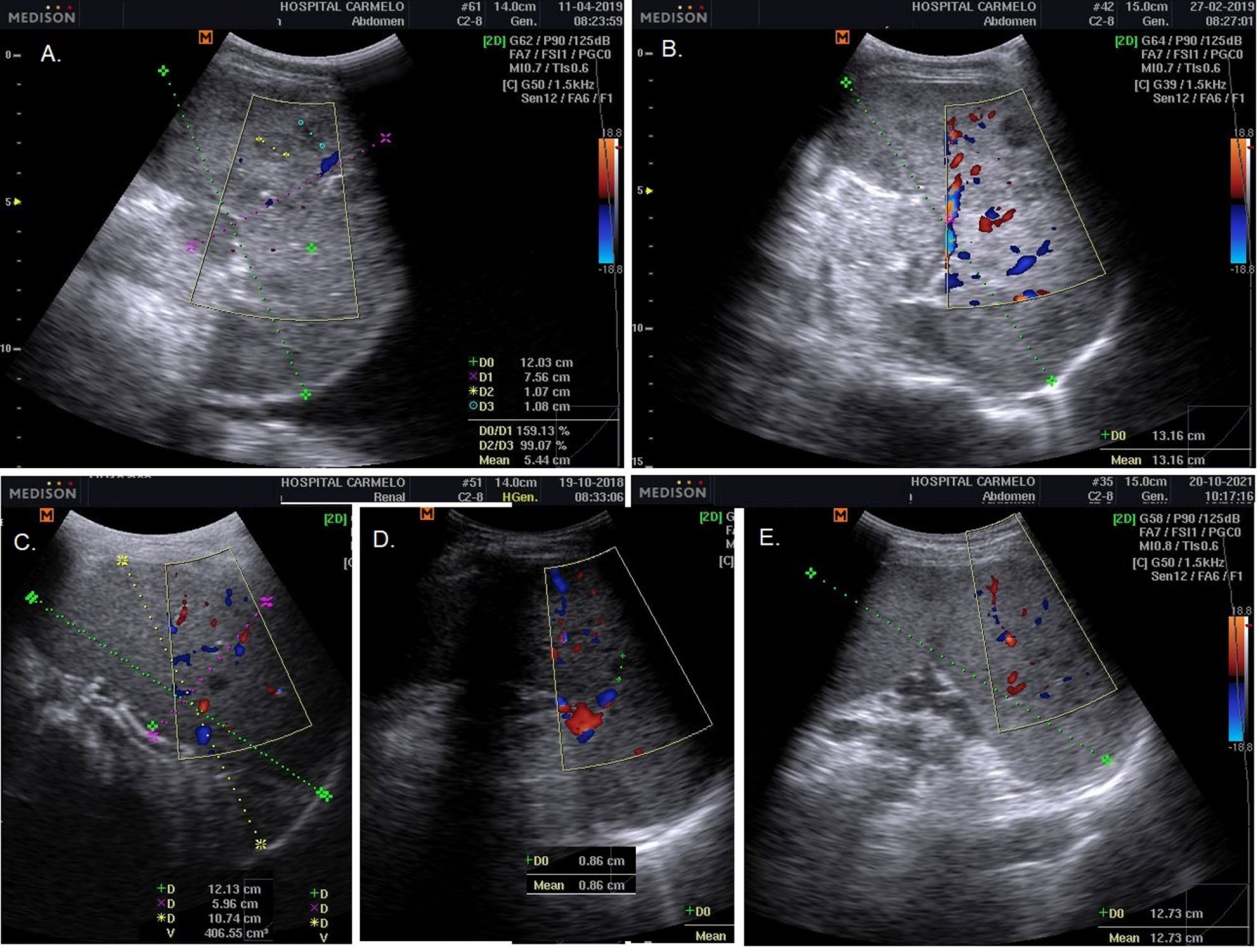
Splenic TB: macronodular pattern characterized by splenomegaly with single or multiple, hypo(anechoic) macronodular lesion; color doppler with hypo flow, compatible with focal caseous splenic lesion (**A**–**C**); and miliary pattern characterized by a splenomegaly with hypo(anechoic) diffuse multiple nodular lesions (**D**, **E**).

*Abdominal tuberculous with splenic involvement* The diagnosis of splenic involvement in TB was classified into either: (1) a miliary pattern characterized by a splenomegaly with hypo(anechoic) diffuse multiple nodular lesions; or (2) a macronodular pattern characterized by splenomegaly with single or multiple, hypo(anechoic) macronodular lesion; color doppler with hypo flow, compatible with focal caseous splenic lesion^18^ (Fig. 6).

**Figure 7:**
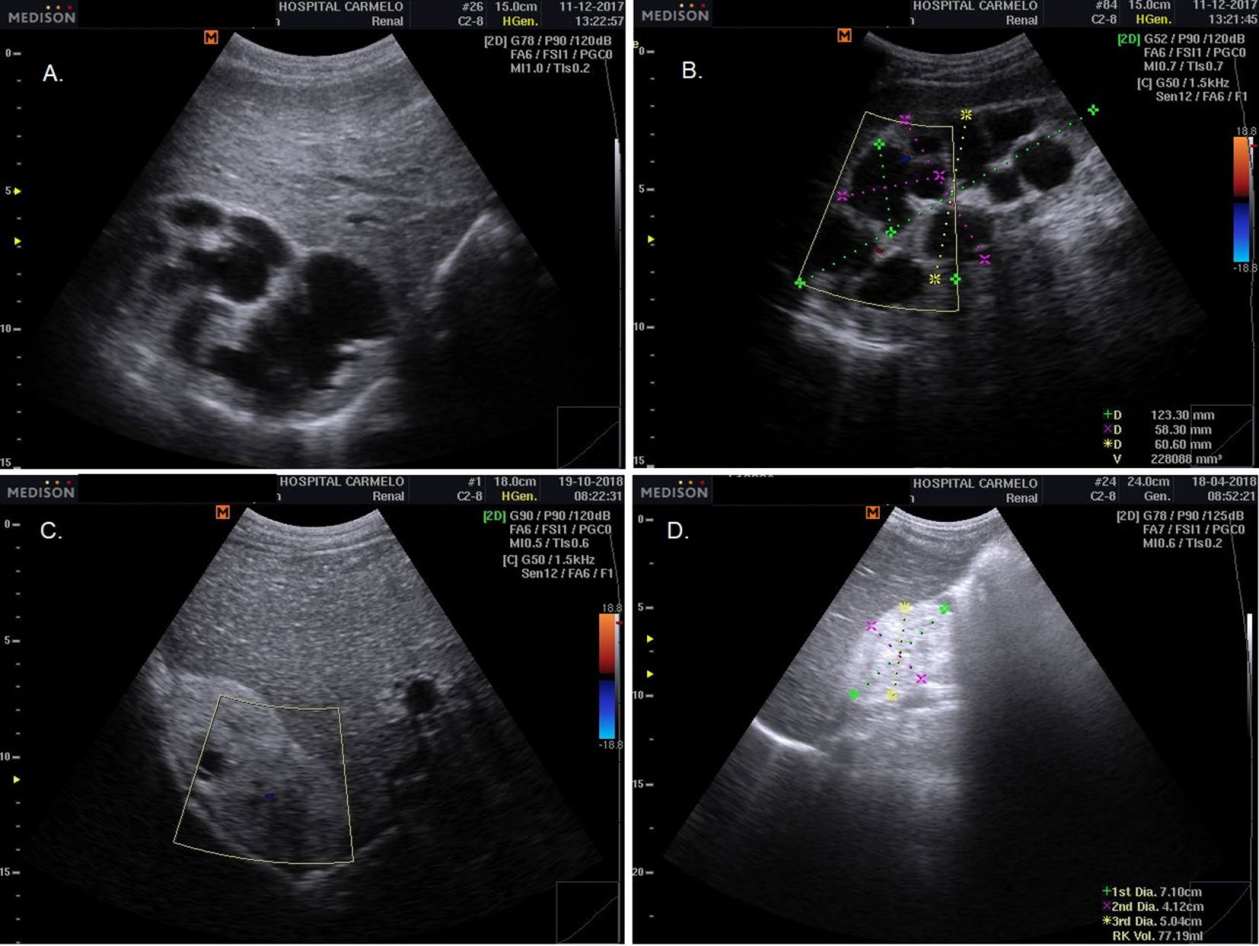
Renal TB: hydrops pattern characterized by nephromegaly with single or multiple hypo(anechoic) rounded lesions and some calcification; hydronephrosis marked by the presence of irregular caliectasis and dilated renal pelvis, caused by varying degrees of fibrosis and obstruction affecting different sites of the urinary tract (e.g., thickening of the ureter wall) (**A**, **B**); and mixed pattern characterized by pathological changes in the kidney such as: similar POCUS findings of chronic kidney failure, hydrops, caseous cavity, fibrosis, pyonephrosis, calcification, inflammation, and atrophy (**C**, **D**).

*Abdominal tuberculous with renal involvement* The diagnosis of renal involvement in TB was classified as having either: (1) a hydrops pattern, characterized by nephromegaly with single or multiple hypo(anechoic) rounded lesions with some calcification^21^, or hydronephrosis, marked by the presence of irregular caliectasis and dilated renal pelvis, caused by varying degrees of fibrosis and obstruction affecting different sites of the urinary tract (e.g., compatible with thickening of the ureter wall)^22^; or (2) a mixed pattern, characterized by pathological changes in the kidney, such as similar POCUS findings of chronic kidney failure, hydrops, caseous cavity, fibrosis, pyonephrosis, calcification, inflammation, and atrophy^22,23^ (Fig. 7). The Xpert MTB/RIF urine assay for TB confirmation was performed.

All TB-infected patients with POCUS-based diagnosis were treated according to the Mozambican national TB guidelines: treatment for any TB-sensitive case includes rifampin, isoniazid, pyrazinamide, and ethambutol for two months, followed by rifampin and isoniazid for four months^11^. If patients were HIV-positive, they were offered ART according to Mozambican national ART guidelines. The first-line ART regimen includes tenofovir, lamivudine, and either efavirenz or dolutegravir^27^.

### Study sample size

The sample consisted of all eligible patients registered as having EPTB on admission to the CHC; therefore, no sampling calculation criteria were applied.

### Data collection

The study team extracted routine clinical data from paper-based in-patient files and electronic POCUS records. Exposure variables were categorized into four fields: (1) length of stay (date of admission and discharge); (2) demographic profile: gender and age group; (3) clinical features: weight loss, abdominal edema, abdominal tenderness, peripheral adenopathy (enlarged lymph nodes), diarrhea, dyspnea, constipation, ART status at diagnosis of TB, and ART immunovirological response; (4) POCUS findings: cervical adenopathy and supraclavicular adenopathy, axillary adenopathy, pleural effusion, pericardial effusion, para-aortic adenopathy, ascites, hepatomegaly, splenomegaly, nephromegaly, and inguinofemoral adenopathy. Data collected from eligible EPTB patients were anonymized to remove identifying details: each patient was given an alphanumeric code, and the anonymized data were included in the data sheet. Spreadsheet data were exported to SPSS for further data analysis.

### Outcome data and statistical analysis of data

The primary outcome was the incidence of in-hospital mortality over the person-time accrued from the date of admission (study enrollment) to the date of discharge.

Statistical analysis was performed using IBM® Statistical Package for the Social Sciences (SPSS) Statistics Software version 25 (International Business Machines Corporation, IBM corp, Release 2017, https://www.ibm.com/legal/copytrade, USA). Patients’ baseline characteristics, as described above, were compared according to outcomes. We calculated frequencies and proportions for categorical data and presented these results by hospitalization outcome (discharge versus death). Quantitative variables were age (expressed as mean with 95% confidence intervals [CIs]) and length of hospital stay (median, interquartile range [IQR]). We present baseline descriptive results with statistical tests. The incidence of in-hospital mortality was calculated as the number of deaths per 100 days of hospital stay. The in-hospital mortality rate was calculated as the number of patients who died during their hospital stay, divided by the total number of included patients admitted during the study period. Kaplan–Meier analyses were conducted to assess time to death during the hospital stay. We compared the proportion of patients who died according to exposure variables using crude and adjusted Cox regression modeling, reporting adjusted hazard ratios (aHR) with corresponding 95% CIs. Predictors of variables with a p value of less than 0.5 in crude analyses were entered in the multivariate model. Schoenfeld residuals were used to evaluate the assumption of proportional hazards. Length of hospital stay has been found to influence in-hospital mortality and was included in all regression analyses as a time-varying exposure.

### Bioethical considerations

The study protocol was reviewed and approved by Institutional Bioethics Committee for Health of Gaza*.* Permission to perform the research was also obtained from the provincial health directorate of Gaza. Analysis was performed on de-identified, aggregated patient level data. The need for written informed consent was explicitly waived by Institutional Bioethics Committee for Health of Gaza *(IRB0002657—Comité Institucional de Bioética para a Saúde de Gaza, 19/CIBS-Gaza/2021),* due to the retrospective nature of the study. This study complied with the Declaration of Helsinki.

### Ethics approval and consent to participate

The Mozambican National Bioethics Committee for Health (*IRB0002657*, *Comité Institucional de Bioética para a Saúde de Gaza,* 19/CIBS-Gaza/2021). approved this analysis. Analysis was performed on de-identified, aggregated patient level data, and the need for written informed consent was explicitly waived.

## Results

### Clinical and demographic characteristics at EPTB diagnosis

A total of 5263 adult in-patients were screened by POCUS at CHC from January 2016 to December 2020. Of these, 390 (7.4%) presented POCUS findings compatible with EPTB (Fig. 1), and 135 (34.6%) of the 390 died.

Mean age on admission was 37.9 years (95% CI 36.9–39.1); 197 (50.5%) were women, and 136 (34.9%) were aged 35–44 years. Median follow-up was 16 days of hospitalization (IQR 7–33). (Table 1).

**Table 1:** Demographic and clinical characteristics of 390 in-patients with extrapulmonary tuberculosis enrolled at Hospital Carmelo de Chokwe, by mortality outcome, 2016–2020.

	Total		Discharged		Died		p-value*
	N (%)	95% CI	N (%)	95% CI	N (%)	95% CI	
Total	390 (100)		255 (65.4)	60.6–70.0	135 (34.6)	30.0–39.4	
**Demographic profile**							
Gender							0.27
Female	197 (50.5)	45.6–55.5	134 (52.5)	46.4–58.6	63 (46.7)	38.4–55.1	
Male	193 (49.5)	44.5–54.4	121 (47.5)	41.4–53.6	72 (53.3)	44.9–61.6	
Age, mean (95% CI)	37.9	36.9–39.1	37.93	36.38–39.48	37.72	35.72–39.72	0.98
Age group							> 0.99
15–24 years	44 (11.3)	8.4–14 .7	28 (11.0)	7.6–15.3	16 (11.9%)	7.2–18.1	
25–34 years	124 (31.8)	27.3–36.5	80 (31.4)	25.9–37.3	44 (32.6)	25.1–40.8	
35–44 years	136 (34.9)	30.3–39.7	90 (35.3)	29.6–41.3	46 (34.1)	26.1–42.3	
45–54 years	44 (11.3)	8.4–14.7	29 (11.4)	7.9–15.7	15 (11.1)	6.6–17.2	
55–64 years	28 (7.2)	4.9–10.1	18 (7.1)	4.4–10.7	10 (7.4)	3.9–12.7	
≥ 65 years	14 (3.6)	2.1–5.8	10 (3.9)	2.0–6.8	4 (3.0)	1.0–6.9	
**Clinical symptoms**							
Weight loss	347 (89.0)	85.6–91.8	225 (88.2)	83.9–91.8	122 (90.4)	84.5–94.5	0.52
Abdominal swelling	42 (10.8)	8.0–14.1	29 (11.4)	7.9–15.7	13 (9.6)	5.5–15.5	0.60
Abdominal tenderness	58 (14.9)	11.6–18.7	31 (12.2)	8.6–16.6	27 (20.0)	13.9–27.3	0.038
Peripheral adenopathy	54 (13.8)	10.7–17.5	35 (13.7)	9.9–18.3	19 (14.1)	9.0–20.7	0.92
Diarrhea	213 (54.6)	49.7–59.5	130 (51.0)	44.9–57.1	83 (61.5)	53.1–69.4	0.048
Dyspnea	72 (18.5)	14.9–22.5	58 (22.7)	17.9–28.2	14 (10.4)	6.1–16.3	0.003
Constipation	12 (3.1)	1.7–5.2	6 (2.4)	1.0–4.8	6 (4.4)	1.9–8.9	0.26
**ART status at TB diagnosis**							0.016
HIV negative	42 (10.8)	8.0–14.1	35 (13.7)	9.9–18.3	7 (5.2)	2.3–9.9	
Pre-ART period	64 (16.4)	13.0–20.3	46 (18.0)	13.7–23.1	18 (13.3)	8.4–19.8	
ART > 90 days	272 (69.7)	65.1–74.1	165 (64.7)	58.7–70.4	107 (79.3)	71.8–85.4	
ART < 90 days	12 (3.1)	1.7–5.2	9 (3.5)	1.8–6.3	3 (2.2)	0.6–5.8	
**Immunvirological response to ART**							
ART immunvirological response							
HIV negative	42 (10.8)	8.0–14.1	35 (13.7)	9.9–18.3	7 (5.2)	2.3–9.9	< 0.001
Optimal immunovirological response	47 (12.1)	9.1–15.6	43 (16.9)	12.7–21.8	4 (3.0)	1.0–6.9	
Immunological non-responder	77 (19.7)	16.0–23.9	45 (17.6)	13.3–22.7	32 (23.7)	17.1–31.4	
Immunovirological failure	160 (41.0)	36.2–46.0	86 (33.7)	28.1–39.7	74 (54.8)	46.4–63.0	
Pre-ART period	64 (16.4)	13.0–20.3	46 (18.0)	13.7–23.1	18 (13.3)	8.4–19.8	

Pearson chi-square; *ART* antiretroviral therapy, *CI* confidence interval, *HIV* human immunodeficiency virus, *TB* tuberculosis.

Table 1 shows patients’ clinical features: 89% had weight loss, 54.6% diarrhea, 18.5% dyspnea, 14.9% abdominal tenderness, 13.8% peripheral adenopathy, 10.8% abdominal swelling, and 3.1% constipation. At baseline, 10.8% were HIV negative. More than two-thirds were HIV-positive and had been on ART for more than 90 days (69.7%), and less than half (41%) had immunovirological failure (Table 1).

Four types of POCUS approaches were used. According to the upper peripheral approach: 12.1% of patients had scrofula; and 1.5%; lower peripheral approach showed inguinofemoral adenopathy. The thoracic approach showed 4.6%, axillary lymphadenopathy; pleural effusion (11.3%), pericardial effusion (11.3%), and pericardial thickness (2.1%); and the abdominal approach showed intra-abdominal lymphadenopathy (73.6%), hepatomegaly (45.9%), ascites (10.8%), splenomegaly (6.2%), and nephromegaly (6.2%) (Table 2).

**Table 2:** POCUS findings in 390 in-patients with extrapulmonary tuberculosis enrolled at Hospital Carmelo de Chokwe (2016–2020), by mortality outcome.

Types of POCUS approaches	Lines of ultrasound view	Total (N = 390)		Discharged (N = 255)		Died (N = 135)		p value*
		N (%)	95% CI	N (%)	95% CI	N (%)	95% CI	
Upper peripheral approach	**Neck and supraclavicular line, thorax**							
	Neck adenopathy (Scrofula)	47 (12.1)	9.1–15.6	31 (12.2)	8.6–16.6	16 (11.9)	7.2–18.1	0.93
Thoracic approach	**Axillary line, thorax**							
	Axillary adenopathy	18 (4.6)	2.9–7.0	13 (5.1)	2.9–8.3	5 (3.7)	1.4–7.9	0.53
	Pleural effusion							0.051
	No abnormalities	341 (87.4)	83.9–90.4	216 (84.7)	79.9–88.7	125 (92.6)	87.3–96.1	
	Pleural effusion	44 (11.3)	8.4–14.7	36 (14.1)	10.3–18.8	8 (5.9)	2.8–10.9	
	Empyema	5 (1.3)	0.5–2.8	3 (1.2)	0.3–3.1	2 (1.5)	0.3–4.7	
	**Epigastric angle**							
	Pericardiac effusion							0.036
	No abnormalities	338 (86.7)	83.0–89.8	214 (83.9)	79.0–88.0	124 (91.9)	86.3–95.6	
	Pericardial effusion	44 (11.3)	8.4–14.7	33 (12.9)	9.2–17.5	11 (8.1)	4.4–13.7	
	Pericardial thickness	8 (2.1)	1.0–3.8	8 (3.1)	1.5–5.8	0 (0.0)		
Abdominal approach	**Mesogastric line**							
	Intra-abdominal adenopathy	287 (73.6)	69.1–77.8	179 (70.2)	64.4–75.6	108 (80.0)	72.7–86.1	0.037
	**Axillary line, abdomen**							
	Ascites	42 (10.8)	8.0–14.1	29 (11.4)	7.9–15.7	13 (9.6)	5.5–15.5	0.60
	Hepatomegaly	179 (45.9)	41.0–50.9	126 (49.4)	43.3–55.5	53 (39.3)	31.3–47.7	0.056
	Splenomegaly							0.52
	No abnormalities	366 (93.8)	91.1–95.9	238 (93.3)	89.8–95.9	128 (94.8)	90.1–97.7	
	Splenomegaly	7 (1.8)	0.8–3.5	6 (2.4)	1.0–4.8	1 (0.7)	0.1–3.4	
	Splenomegaly with focal hypoechoic lesion	17 (4.4)	2.7–6.7	11 (4.3)	2.3–7.3	6 (4.4)	1.9–8.9	
	Kidney abnormalities							0.013
	No abnormalities	366 (93.8)	91.1–95.9	245 (96.1)	93.2–98.0	121 (89.6)	83.7–93.9	
	Hydrops pattern	8 (2.1)	1.0–3.8	5 (2.0)	0.8–4.2	3 (2.2)	0.6–5.8	
	Mixed pattern	16 (4.1)	2.5–6.4	5 (2.0)	0.8–4.2	11 (8.1)	4.4–13.7	
Lower peripheral approach	Inguinofemoral adenopathy	6 (1.5)	0.6–3.1	4 (1.6)	0.5–3.7	2 (1.5)	0.3–4.7	0.95

*Pearson chi-square.

### In-patient mortality with EPTB

Overall, 135 (34.6%, 95% CI 30.0–39.4) of the 390 in-patients with EPTB died. Among these, 53.3% (95% CI 44.9–61.6) were men. The proportion of patients who died before discharge was significantly higher in those reporting abdominal tenderness (20% [95% CI 13.9–27.3] vs 12.2% [95% CI 8.6–16.6] p = 0.038) or diarrhea (61.5% [95% CI 53.1–69.4] vs 51% [95% CI 44.9–57.1], p = 0.048); in patients on ART for more than 90 days (79.3% [95% CI 71.8–85.4] vs 64.7% [95% CI 58.7–70.4], p = 0.016) or presenting immunovirological failure on ART (54.8% [95% CI 46.4–63.0] vs 33.7% [95% CI 28.1–39.7], p = 0.000; Table 1); and in those with a mixed pattern on kidney POCUS (8.1% [95% CI 4.4–13.7] vs 2.0% [95% CI 0.8–4.2], p = 0.013; Table 2).

### Risk and predictors of death on ART

Overall, the 390 in-patients with EPTB with POCUS features contributed a total of 6240 person-days to the study data. The overall mortality rate was 2.16 per 100 person-days (95% CI 1.73–2.31). According to the multivariable Cox regression model, having an optimal immunovirological response to ART conferred an 88% lower risk of death (aHR 0.12, 95% CI 0.04–0.35, p < 0.001) compared to being HIV-negative (Table 3).

**Table 3:** Cox proportional hazards model for mortality in 390 in-patients with extrapulmonary tuberculosis, Carmelo Hospital of Chókwè (Mozambique), 2016–2020.

	Died N (%)	PDH	Incidence per 100 PDH (95% CI)	Crude HR (95% CI)	p value	Adjusted HR (95% CI)	p value
Total	135 (34.6)	6240	2.16 (1.73–2.31)				
**Demographic profile**							
Gender							
Female	63 (46.7)	3349	1.88 (1.45–2.13)	Ref.		Ref.	
Male	72 (53.3)	2895	2.49 (1.96–2.87)	1.25 (0.89–1.75)	0.20	1.71 (1.17–2.49)	0.005
Age group							
15–24 years	16 (11.9)	748	2.14 (1.52–3.64)	Ref.		Ref.	
25–34 years	44 (32.6)	2418	1.82 (1.36–2.37)	0.89 (0.50–1.58)	0.70	0.90 (0.48–1.66)	0.73
35–44 years	46 (34.1)	2040	2.25 (1.69–2.42)	0.98 (0.55–1.73)	0.94	1.00 (0.54–1.84)	0.99
45–54 years	15 (11.1)	968	1.55 (0.95–1.70)	0.82 (0.41–1.67)	0.59	0.68 (0.32–1.44)	0.32
55–64 years	10 (7.4)	350	2.86 (2.10–5.10)	1.09 (1.49–2.39)	0.84	0.83 (0.35–1.97)	0.68
≥ 65 years	4 (3.0)	133	3.01 (1.36–4.76)	0.98 (0.33–2.93)	0.97	1.86 (0.53–6.48)	0.33
**Clinical symptoms**							
Clinical features							
Weight loss							
No	13 (9.6)	645	2.02 (1.37–3.02)	Ref.		Ref.	
Yes	122 (90.4)	5899	2.07 (1.76–2.34)	1.07 (1.60–1.90)	0.82	0.63 (0.11–3.58)	0.60
Abdominal swelling							
No	122 (90.4)	5568	2.19 (1.75–2.34)	Ref.		Ref.	
Yes	13 (9.6)	651	2.00 (1.47–3.44)	0.95 (0.53–1.68)	0.85	0.96 (0.19–4.96)	0.96
Abdominal tenderness							
No	108 (80.0)	5976	1.81 (1.55–2.03)	Ref.		Ref.	
Yes	27 (20.0)	551	4.90 (2.74–6.65)	1.69 (1.11–2.59)	0.015	1.68 (1.00–2.82)	0.050
Diarrhea							
No	52 (38.5)	2655	1.96 (1.47–2.10)	Ref.		Ref.	
Yes	83 (61.5)	3621	2.29 (1.86–2.78)	1.24 (1.88–1.76)	0.22	1.31 (0.14–12.4)	0.82
Dyspnea							
No	121 (89.6)	5088	2.38 (1.90–2.72)	Ref.		Ref.	
Yes	14 (10.4)	1368	1.02 (0.81–1.62)	0.50 (0.29–0.87)	0.014	0.75 (0.26–2.14)	0.59
Constipation							
No	129 (95.6)	6237	2.07 (1.71–2.28)	Ref.		Ref.	
Yes	6 (4.4)	78	7.69 (1.72–16.67)	1.91 (1.84–4.33)	0.12	1.14 (0.29–4.58)	0.85
**ART status at TB diagnosis**							
HIV negative	7 (5.2)	462	1.52 (0.93–2.08)	Ref.		Ref.	
Pre-ART period	18 (13.3)	1120	1.61 (1.28–2.16)	1.54 (1.64–3.69)	0.33	2.13 (0.80–5.68)	0.13
ART > 90 days	107 (79.3)	4624	2.31 (1.87–2.62)	2.12 (2.99–4.57)	0.055	4.03 (1.50–10.78)	0.006
ART < 90 days	3 (2.2)	330	0.91 (0.46–4.17)	1.11 (1.29–4.31)	0.88	2.59 (0.53–12.70)	0.24
**Immunovirological response to ART**							
HIV negative	7 (5.2)	462	1.52 (0.93–2.08)	Ref.		Ref.	
Optimal immunovirological response	4 (3.0)	1034	0.39 (0.26–0.45)	0.39 (0.11–1.34)	0.14	0.12 (0.04–0.35)	< 0.001
Immunological non-responders	32 (23.7)	1232	2.60 (1.81–2.77)	2.26 (2.00–5.13)	0.051	0.80 (0.50–1.28)	0.35
Immunovirological failure	74 (54.8)	2480	2.98 (2.20–4.63)	2.55 (2.17–5.54)	0.018	(not estimated)	
Pre–ART Period	18 (13.3)	1120	1.61 (1.28–2.16)	1.53 (1.64–3.68)	0.34	(not estimated)	

POCUS findings							
**Upper peripheral approach**							
1. Neck and supraclavicular line, thorax							
Neck adenopathy (scrofula)							
No	119 (88.1)	5488	2.17 (1.73–2.48)	Ref.		Ref.	
Yes	16 (11.9)	705	2.27 (1.31–2.62)	1.03 (1.61–1.74)	0.91	1.12 (0.37–3.41)	0.84
**Thorax approach**							
2. Axillary line, thorax							
Axillary adenopathy							
No	130 (96.3)	5952	2.18 (1.75–2.33)	Ref.		Ref.	
Yes	5 (3.7)	351	1.42 (0.99–2.14)	0.74 (0.30–1.81)	0.51	0.80 (0.27–2.75)	0.69
Pleural effusion							
No abnormalities	125 (92.6)	5456	2.29 (1.83–2.44)	Ref.		Ref.	
Pleural effusion	8 (5.9)	836	0.96 (0.79–2.02)	0.52 (0.25–1.06)	0.072	0.67 (0.25–1.75)	0.41
Empyema	2 (1.5)	165	1.21 (0.31–6.67)	0.84 (0.21–3.47)	0.82	1.30 (0.23–7.19)	0.77
3. Epigastric angle							
Pericardiac effusion							
No abnormalities	124 (91.9)	5070	2.45 (2.04–2.62)	Ref.		Ref.	
Pericardial effusion	11 (8.1)	1012	1.09 (0.86–1.56)	0.61 (0.33–1.14)	0.12	0.79 (0.34–1.85)	0.56
Pericardial thickness	0 (0.0)	160	0.00 (0.00–0.00)	0.00 (0.00–8.41)	0.96	0. 00 (0.00–1.98)	0.96
**Abdominal approach**							
4. Mesogastric line							
Intra-abdominal adenopathy							
No	27 (20.0)	2060	1.31 (1.09–1.75)	Ref.		Ref.	
Yes	108 (80.0)	4592	2.35 (1.88–2.69)	1.38 (1.90–2.11)	0.14	0.75 (0.24–2.28)	0.61
5. Axillary line, abdomen							
Ascites							
No	122 (90.4)	5568	2.19 (1.75–2.34)				
Yes	13 (9.6)	651	2.00 (1.47–3.44)	0.95 (0.53–1.68)	0.85	Not estimated	
Hepatomegaly							
No	82 (60.7)	3587	2.29 (1.85–2.78)	Ref.		Ref.	
Yes	53 (39.3)	2685	1.97 (1.41–2.11)	0.82 (0.58–1.16)	0.26	1.44 (0.19–10.73)	0.72
Splenomegaly							
No abnormalities	128 (94.8)	5856	2.19 (1.75–2.33)	Ref.		Ref.	
Splenomegaly	1 (0.7)	105	0.95 (0.32–7.14)	0.47 (0.07–3.36)	0.45	0.42 (0.05–3.66)	0.43
Splenomegaly with focal hypoechoic lesion	6 (4.4)	289	2.08 (0.90–3.92)	0.93 (0.41–2.12)	0.87	0.44 (0.14–1.43)	0.17
Kidney abnormalities							
No abnormalities	121 (89.6)	5856	2.07 (1.65–2.20)	Ref.		Ref.	
Hydrops pattern	3 (2.2)	120	2.50 (0.71–18.75)	1.19 (1.38–3.74)	0.77	1.75 (0.41–7.48)	0.45
Mixed pattern	11 (8.1)	256	4.30 (2.64–6.88)	2.24 (2.21–4.16)	0.01	2.91 (1.38–6.10)	0.005
**Lower peripheral approach**							
Inguinofemoral adenopathy							
No	133 (98.5)	6144	2.16 (1.73–2.31)	Ref.		Ref.	
Yes	2 (1.5)	216	0.93 (0.68–2.22)	0.78 (0.19–3.15)	0.73	1.31 (0.26–6.48)	0.74

*PDH* person-days of hospitalization, *ART* antiretroviral treatment, *HR* hazard ratio, *CI* confidence interval.

Men had nearly twice the risk of death compared to women (aHR 1.71, 95% CI 1.17–2.49, p = 0.005), and people with abdominal tenderness had nearly double the risk compared to those without this complaint (aHR 1.68, 95% CI 1.00–2.82, p = 0.050). Being on ART for more than 90 days at baseline was associated with a four-fold higher risk of death compared to those who were HIV-negative (aHR 4.03, 95% CI 1.50–10.78, p = 0.006). Those who had mixed patterns on kidney POCUS had nearly three times the risk of death compared to those with a normal appearance (aHR 2.91, 95% CI 1.38–6.10, p = 0.005; Table 3).

In-hospital mortality was not significantly associated with age group (demographic variable); weight loss, abdominal swelling, diarrhea, dyspnea, or constipation (clinical); or the POCUS findings of peripheral adenopathy (neck, axillary and inguinofemoral), pleural effusion (and/or empyema), pericardial effusion (and/or pericardial thickness), intra-abdominal lymphadenopathy, hepatomegaly, or splenomegaly (with macronodular or miliary pattern) (Table 3).

### Cumulative mortality rate

**Figure 8:**
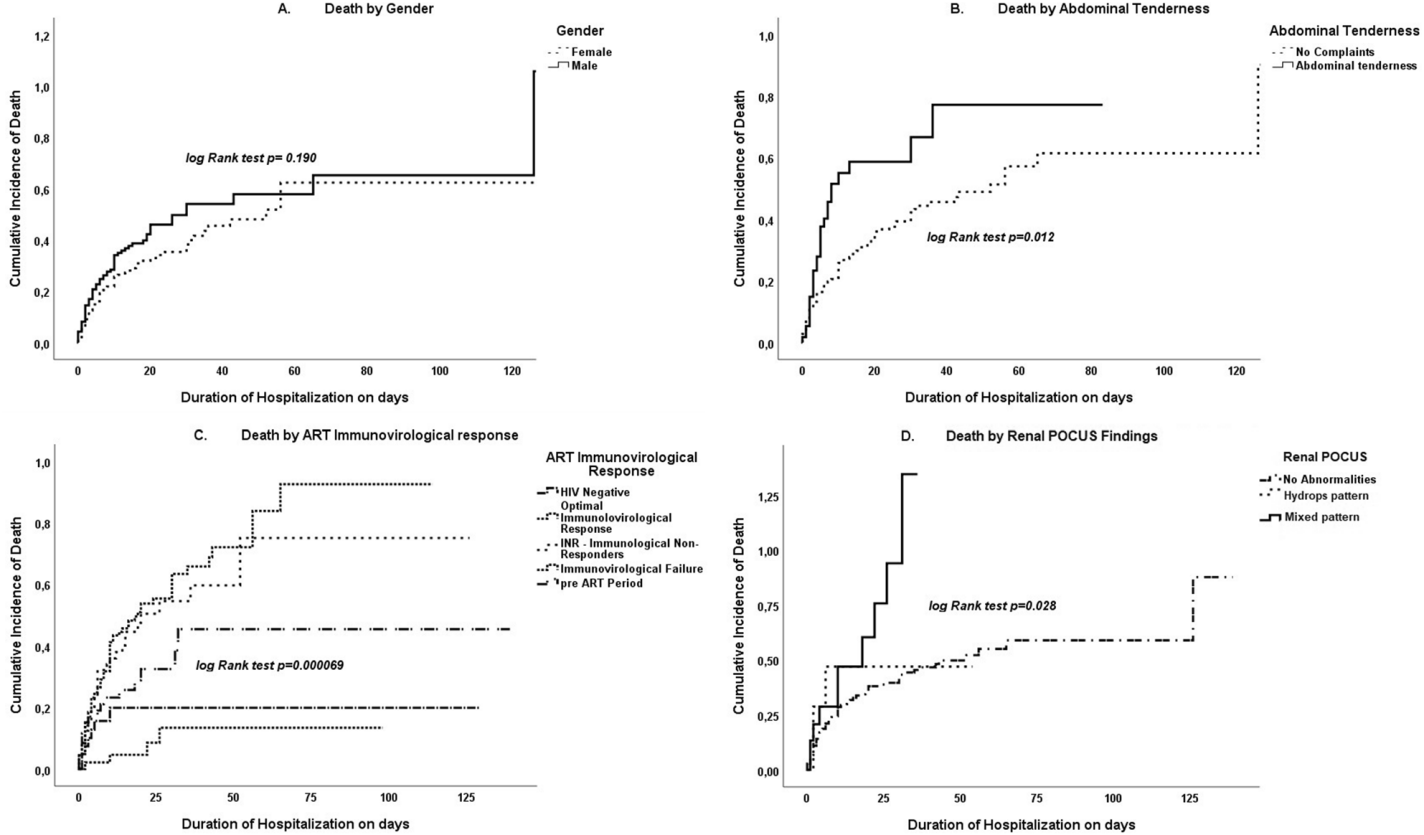
Kaplan–Meier plot for 390 in-patients with extrapulmonary tuberculosis at Carmelo Hospital of Chókwè (2016–2020) by: (**A**) gender, (**B**) abdominal tenderness symptoms, (**C**) antiretroviral treatment (ART) immunovirological response, and (**D**) renal POCUS findings.

Figure 8 presents the cumulative mortality rate by gender, abdominal tenderness symptoms, ART immunovirological response, and nephromegaly POCUS findings. Men had a higher mortality rate—above 40%—after 16 days of follow-up, compared to 30% in women (log-rank test p = 0.190; Fig. 8A). Those who reported abdominal tenderness as their chief complaint had a higher cumulative mortality rate, of more than 59%, after 16 days of follow-up (vs 32% of those without abdominal tenderness, log-rank test p = 0.012; Fig. 8B). Immunological failure to ART and being an immunological non-responder were also variables associated with higher cumulative mortality, at 57% and 56%, respectively after 16 days of follow-up (versus HIV-negative, 3%, and optimal immunovirological response, 1%, log-rank p < 0.001; Fig. 8C). Having a mixed pattern on kidney POCUS was associated with higher cumulative mortality rate, which exceeded 48% at 16 days of follow-up (vs 23% in those with normal renal appearance, log-rank p = 0.028; Fig. 8D).

## Discussion

This is one of the first hospital-based retrospective cohort studies that simultaneously analyzes POCUS and in-hospital death in EPTB-infected patients. We aimed to determine whether the sonographic features on POCUS were associated with in-hospital death during TB care in newly diagnosed EPTB in-patients in Chókwè district. Like other studies in Eastern and Southern Africa, we observed a marked improvement in early detection of EPTB cases after POCUS implementation^24,25^. Multiple organ involvement of TB was also found, with EPTB invading more than one anatomic organ, as in pluri-visceral TB disease (e.g., lymph nodes plus spleen and/or kidney) and pluri-cavity TB disease (e.g., peritoneal plus pleura and/or pericardial effusion)^2,18,28^.

POCUS is the most efficient to detect primarily Abdominal TB (abdominal lymphadenopathy, splenic microabscess, ascites), then to screen for pleural TB and pericardial TB^24,29^. It is relatively useless to detect other forms of EPTB (such as meningeal or central nervous system TB, osteoarticular TB)^30^.

The data of the present study also shown a marked shift in the patterns of EPTB diagnosis following POCUS implementation.

Recent studies in Eastern and Southern Africa have also shown that the FASH algorithm (focused assessment with sonography for HIV/TB), quite similar to POCUS approach carried on by us, accelerated early detection and treatment of EPTB, without eliminating other TB diagnostic analyses (Xpert MTB/RIF assay, urine LAM, histological, and chest X-ray) previously offered to in-patients. Yet, our health facilities began to shorten the time to TB treatment immediately after POCUS implementation^25,31^.

The present study showed nearly 9 of every 10 in-patients reported weight loss as the main complaint, associated or not with other symptoms such as: diarrhea, dyspnea, abdominal tenderness, abdominal swelling, superficial adenopathy, and constipation. These results are quite similar to other reports from the region^25^.

The POCUS was an indispensable imaging test to diagnose anatomic organ-specific EPTB based on the patients’ chief complaints. Therefore, a clearly defined POCUS view was undertaken, from superficial lymph node view to thorax and/or abdominal views, in line with in-patients’ main complaint^2^.

In the general POCUS findings observed in this study, the abdominal approach showed more abnormalities compatible with EPTB diagnosis compared to the chest and/or superficial lymph nodes approach. Almost three-quarters of in-patients showed intra-abdominal lymphadenopathy, associated or not with ascites, hepatomegaly, splenomegaly and nephromegaly. These results are similar to other studies revealing intra-abdominal lymphadenopathy as the main sonographic finding, strongly associated with abdominal TB^18,25^, suggesting the urgent need to support integrated POCUS for those with suspected TB.

Those who had an optimal immunovirological response to ART in our hospitalized cohort showed a lower risk of death. This result highlights the critical importance of adhering to ART and strengthening psychosocial support systems for HIV testing and ART treatment^32^.

Similar results from South Africans also report HIV co-infected men was associated with increased odds of death (AOR 2.4; CI 2.1–2.8) compared to HIV co-infected women (AOR: 1.9; CI 1.7–2.1)^33^. This may be due to the following reason, men prioritize their work to ensure food security and maintenance of masculinity norms, consequently delaying seeking and engagement in TB care ^34^. Therefore, it is necessary to strengthen interventions that leverage men's social networks based on existing resources, that promote male involvement in the TB care cascade, and thus improve TB treatment outcomes^34^.

Several reports similar to the present study showed that having abdominal tenderness as the chief complaint was associated with a higher risk of death^35,36^. Thus, abdominal tenderness must be considered a sign of a worsening clinical picture, as any TB-infected abdominal viscera can trigger peritonitis ^37^. Although TB peritonitis is a surgical emergency, it is rarely performed due to the frailty of hospitalized patients, which is aggravated by severe immunosuppression in people with TB/HIV co-infection^38^.

Our research showed that the use of ART for more than three months was associated with a higher risk of death, particularly in those with immunovirological failure or immunological non-responders to ART. These findings are in keeping with studies from other low-income settings, which report the negative impact that a low CD4 cell count during the TB treatment initiation period has on mortality outcomes^32^. This is why early detection of viral load and initial CD4 counts are recommended for all TB/HIV co-infected patients, along with urgent switching to second-line ART in those with immunovirological failure^39^.

The finding of a mixed pattern on kidney POCUS was associated with a higher risk of death in our patients. These results agree with the literature, which underlines the role of disease masking and delayed diagnosis (due to vague clinical features and a low index of suspicion) in disease progression, irreversible tissue and organ damage, and chronic kidney failure. This impact is compounded by persistent challenges in performing hemodialysis and kidney transplantation in low-resource settings^18,40,41^.

Additionally, a miliary pattern splenomegaly on POCUS was strongly associated with pulmonary miliary pattern on chest X-ray, suggesting an urgent need to perform POCUS for all TB-suspected patients, histologically confirmed or not, particularly in those with suggestive TB on chest X-ray in order to rule out EPTB^18,25^. POCUS stands out as a valuable tool to promptly identify EPTB involvement, even in those who were previously diagnosed as PTB.

Although there are more cases of isolated PTB than EPTB, the latter carries a higher risk of mortality, particularly in TB/HIV-coinfected patients with severe immunosuppression^42,43^.

The main strength of this study is its novelty: it is the first study in Eastern or Southern Africa to evaluate the association between point-of-care ultrasound findings and the risk of death in in-patients with EPTB.

The study also has some limitations. First, it was a retrospective cohort analysis, so data were limited to recorded variables. Second, the study period does not define the exact date of the end of the EPTB treatment, only the date of discharge from hospital. Third, it did not discriminate between new and recurrent cases of EPTB. Fourth, only patients diagnosed during hospitalization were included. Fifth, the CHC is a referral hospital for TB, receiving critically ill patients with very advanced disease, which may explain the high mortality rate in this health facility. Thus, our results do not reflect the results of EPTB mortality across the country. Considering the resource limitations in the public sector, death estimates across the national health service may be higher.

## Conclusion

EPTB is a disease that can manifest in an isolated or multiorgan form and contributes greatly to tuberculosis-associated mortality in HIV-immunosuppressed patients. Variables associated with an increased risk of death were male gender, abdominal pain, ART for more than three months (with immunovirological failure or non-response to ART) and having a mixed pattern of kidney POCUS characteristics. Early detection of these risk factors may have a direct impact in reducing TB mortality, and the POCUS approach to diagnosing EPTB provides a simple, feasible and affordable intervention in resource-limited settings like Mozambique. Investment in and implementation of diagnostic algorithms that integrate POCUS examinations are highly recommended.

## Data Availability

The datasets analyzed during the current study are not publicly available but are available from the corresponding author on reasonable request.

## Abbreviations

aHR: Adjusted hazard ratios
ART: Antiretroviral treatment
CI: Confidence intervals
CHC: Carmelo Hospital of Chókwè
EPTB: Extrapulmonary tuberculosis
ESA: Eastern and Southern Africa
FASH: Focused assessment with sonography for HIV/TB
IQR: Interquartile range
LAM: Mycobacterial lipoarabinomannan
NTP: Mozambican National TB Program
PHC: Primary healthcare clinic
POCUS: Point-of-care ultrasound
